# Depression treatment patterns among individuals with osteoarthritis: a cross sectional study

**DOI:** 10.1186/1471-244X-13-121

**Published:** 2013-04-22

**Authors:** Parul Agarwal, Xiaoyun Pan, Usha Sambamoorthi

**Affiliations:** 1 School of Pharmacy, Department of Pharmaceutical Systems and Policy, West Virginia University, Morgantown, WV, USA

**Keywords:** Arthritis, Antidepressants, Psychotherapy, MEPS, Depression treatment

## Abstract

**Background:**

Arthritis and depression often co-occur; however, studies that describe patterns of depression treatment among individuals with arthritis are scant. The purpose of the study was to examine depression treatment patterns among individuals with osteoarthritis (OA) by predisposing, enabling, need factors, personal health practices and external health environment.

**Methods:**

Retrospective cross-sectional design was used. Data were obtained from 2008 and 2010 Medical Expenditure Panel Survey (MEPS). The sample consisted of 647adults aged over 21 years with depression and OA. Depression treatment was categorized as: 1) No treatment;2) antidepressant use only and 3) both antidepressants and psychotherapy (combination therapy). Chi- square tests and multinomial logistic regressions were used to describe patterns of depression treatment. All analysis was performed using Statistical Analysis Software (SAS) version 9.3.

**Results:**

Overall, 13.0% of the study sample reported no depression treatment, 67.8% used antidepressants only and 19.2% used combination therapy. Among individuals with OA significant subgroup differences in depression treatment were observed. For example, African Americans were less likely to report depression treatment compared to whites [antidepressants: AOR=0.33, 95% CI=0.21,0.51; combination therapy: AOR=0.39, 95% CI=0.23, 0.65]. Elderly adults were more likely to receive antidepressants and less likely to receive psychotherapy as compared to younger adults [AOR=0.53, 95% CI= 0.28,0.98]. Adults with anxiety were more likely to report depression treatment compared to those without anxiety [antidepressants: AOR=1.53, 95% CI=1.06, 2.22; combination therapy: AOR=3.52, 95% CI=2.40, 5.15].

**Conclusion:**

Future research needs to examine the reason for low rates of combination therapy as well as subgroup differences in combination therapy among individuals with OA.

## Background

Individuals with arthritis have high rates of co-occurring depression and/or anxiety [1]. Prior studies have reported negative impacts of depression on the quality of life of individuals with rheumatoid arthritis (RA), osteoarthritis (OA) and fibromyalgia [2-4]. Among individuals with arthritis, depression is associated with increased pain, work disability and functional decline [5-7]. While pain is common in individuals with arthritis [8], depression can exacerbate this problem because depression is known to independently cause pain [9]. Similarly, pain can also lead to depression, suggesting that the relationship between depression and pain is bi-directional [10]. Therefore, the management strategies for depression should take into account both depressive symptoms and pain [11].

Antidepressants have been found to be effective in reducing pain among individuals with OA and fibromyalgia [12,13]. Although, antidepressants therapy is the major modality of treatment for depression, it may be used to treat both depression and pain as well for individuals with arthritis [4,13]. For example, a randomized controlled trial (RCT) known as Stepped Care for Affective Disorders and Musculoskeletal Pain found that antidepressant therapy followed by pain self-management program resulted in improvement in depression scores and reduction in pain severity and disability [14].

Combination therapy (antidepressants with psychotherapy) is also found to be effective in treating depression [15]. American Psychiatric Association recommends combination of psychotherapy and antidepressant medication as initial treatment for individuals with moderate to severe major depressive disorder [15]. In an RCT of 681 adults with chronic major depressive disorder it was concluded that a combination of nefazodone (an antidepressant) and psychotherapy was more effective in relieving depression compared to either form of treatment alone [16]. This finding has also been substantiated among individuals with arthritis. Results from an RCT performed at 18 primary care clinics - IMPACT (Improving Mood Promoting Access to Collaborative Treatment) among 1,801 older adults with arthritis (93% with OA) and depression, revealed that individuals who received combination therapy (antidepressants with psychotherapy) experienced not only a reduction of depressive symptoms and pain but also improvements in the functional status and quality of life compared to those who received usual depression treatment [4].

### Objective

The aforementioned studies suggest that antidepressants and psychotherapy can provide relief from depression and pain among individuals with OA. However, it is not known whether findings from these clinical trials have been translated into routine clinical practices of depression care among individuals with OA in real-world settings. In fact, research on patterns of depression treatment among various subgroups of individuals with depression and OA is sparse. Such studies are critical for understanding subgroup differences in depression treatment as well as informing intervention efforts to promote effective depression care. Therefore, the main objective of this study was to examine patterns of depression treatment by predisposing, enabling, need factors, personal health practices and external health environment among individuals with OA.

### Conceptual framework

The basic framework for the study is the expanded behavioral model on use of health services proposed by Anderson [17]. The model posits that healthcare treatment or use is affected by: (1) each individual’s unique predisposition for using services (predisposing factors), (2) the means available to each individual for obtaining services (enabling factors), and (3) each individual’s level of need (need factors). Under this model predisposing variables (e.g. gender, age, and race), enabling variables (e.g. marital status, education and poverty status), need variables (e.g. health status variables and pain), personal health practices (e.g. physical activity, obesity and smoking) and external healthcare environment (e.g. metro status) affect depression treatment.

## Methods

### Study design

Retrospective cross-sectional study design was used to describe patterns of depression treatment among individuals with OA.

### Data source

Data were obtained from two years (2008 and 2010) of the Medical Expenditure Panel Survey (MEPS) dataset, which is available for public use. MEPS is a large-scale nationally representative survey of U.S. families and individuals, medical providers and employers, implemented by the Agency for Healthcare Research and Quality (AHRQ). MEPS contains information on medical conditions and mental health conditions, healthcare use services and medical and mental health treatment including prescription drugs and counseling [18]. To minimize underreporting of conditions, MEPS used extensive probes, provided the respondents with calendars, and used shorter recall period [18]. In addition, MEPS elicited information on medical and mental health conditions from medical providers. Respondents were queried whether: 1) they had been diagnosed with specific conditions, 2) the household members had experienced a medical event such as emergency visit or office visit for the condition, 3) the condition caused disability, and 4) the chronic condition bothered the respondent during a specific reference period [19]. The medical conditions reported by the household respondent were found to be consistent with data from medical care providers [20].

Information on medical and mental health conditions was collected verbatim from the household respondents. These health conditions were then converted to be consistent with the International Classification of Diseases, 9th Edition, Clinical Modification (ICD-9-CM) codes by professional coders. These ICD-9-CM codes were then converted by AHRQ into clinical classification codes [21]. Medical condition files available to the researchers contain 3-digit ICD-9-CM and clinical classification codes. For the purpose of analysis, all health conditions used in the paper were based on clinical classification codes or ICD-9-CM codes unless otherwise specified.

### Analytical sample

For the current study, two years of cross-sectional data were pooled to gain an adequate sample size as recommended by MEPS designers [22]. As MEPS follows individuals for two years, data for 2008 and 2010 were used rather than two consecutive years to ensure that repeated observations from the same individual are not included [18]. The analytical sample consisted of adults (n=647) with depression, aged 21 years or older, alive during the observation year and who reported OA. Individuals with OA were identified by an affirmative response to a question that queried whether the respondents have ever been diagnosed with arthritis or if they had clinical classification codes indicating arthritis in their medical conditions file. A small number of individuals who used only psychotherapy for depression treatment were excluded (n = 25). Individuals who reported not having any medical expenditure during the calendar year were also excluded.

### Measures

#### Dependent variable: depression treatment

*Any Antidepressant Use* was identified from prescription drugs file using therapeutic class codes. The MEPS linked drug names and national drug codes to Multum-lexicon classification scheme to classify prescription drugs into various therapeutic classes. These therapeutic class codes were made available to the researchers. In the current study, antidepressants were identified from the therapeutic class code 247. Individuals with one or more prescriptions for antidepressants were considered as antidepressant users.

*Any Psychotherapy Use* was derived from outpatient and office-based provider visits files. These files contained information on reasons for the visits. Individuals with at least one visit with the stated purpose of psychotherapy or mental health counseling were considered to receive psychotherapy [23].

Antidepressant and psychotherapy use were combined to represent combination therapy. Depression treatment was categorized into 3 groups: (1) no depression treatment, (2) antidepressant use only, (3) and psychotherapy with antidepressants (combination therapy).

#### Independent variables

Predisposing variables consisted of gender (women, men), race (white, African Americans, and others) and age in years grouped into 4 categories (22–39, 40–49, 50–64, 65 and above). Enabling factors consisted of marital status (married and not married), education (less than high school, high school, and above high school), poverty status (poor or near poor, middle income, and high income) and health insurance (private, public, and uninsured). Need variables included perceived physical and mental health status (excellent or very good, good, and fair or poor), functional disability (yes/no), pain (pain and no pain), and chronic conditions such as chronic obstructive pulmonary disease (COPD), diabetes, heart disease, and anxiety. Personal health practices included current smoking (current smoker, and others), physical activity (moderate to vigorous 3 times/week versus no physical activity), and body mass index (BMI) categories (under or normal, overweight, and obese). Metro status (metro versus non metro) was used to represent external healthcare environment.

#### Pain

Pain was included as one of the need factors because depression treatment can often be influenced by presence of pain [11]. Antidepressants are sometimes used to relieve pain among individuals with arthritis and other chronic conditions [24]. Information about pain was extracted from a question that queried the respondents whether pain interfered with their normal work outside the home and housework during the past 4 weeks. In MEPS, pain was reported on a 5-point scale: 1) Not at all, 2) A little bit, 3) Moderately, 4) Quite a bit, and 5) extremely. For purposes of this study pain was classified into two categories: 1) pain and 2) no pain. Self-reported pain from MEPS has been used in published literature to estimate cost of pain [25].

### Statistical analysis

Chi-square tests were used to examine significant subgroup differences in depression treatment among individuals with OA. Multinomial logistic regressions were used to examine patterns of depression treatment by predisposing, enabling, need factors, personal health practices and external healthcare environment. The dependent variable consisted of: (1) no depression treatment, (2) antidepressant use only, and (3) psychotherapy with antidepressants (combination therapy). For the dependent variable, “no depression treatment” was used as the reference category. The parameter estimates from the regression were transformed to adjusted odds ratios (AORs) and their corresponding 95% confidence intervals (CIs) were examined. All analyses accounted for complex survey design of MEPS with Statistical Analysis Software (SAS) version 9.3.

## Results

Among individuals with OA and depression the majority were women (76.0%), whites (87.2%), and older adults above age 50 (83.1%). Nearly one-third (34.4%) were below the 100% - 200% federal poverty line. Only 24.4% reported excellent/very good physical health and 33.5% reported excellent/very good mental health. Anxiety was prevalent in 25.1% of the individuals with OA and depression. An overwhelming majority of adults with OA reported pain (87.2%). (Data not presented in tabular form).

Table 1 presents the weighted percent of depression treatment categories by predisposing, enabling, need factors, personal health practices and external health environment. Except for few variables (gender, poverty status, metro status, physical activity, and pain) all subgroup differences were significant at p <0.05. For example, a significantly higher proportion of African Americans (27.8%) reported “No depression treatment” compared to whites (11.1%). Only 7.4% of adults with anxiety reported “no depression treatment” compared to those without anxiety (15.0%).

**Table 1:** Weighted percent of depression treatment among individuals with depression and osteoarthritis: medical expenditure panel survey, 2008 and 2010

		**No treatment**	**Antidepressants only**	**Antidepressants & psychotherapy**	**sig**
		**Wt. (%)**	**Wt. (%)**	**Wt. (%)**	
	**ALL**	**13.0**	**67.8**	**19.2**	
**Predisposing factors**					
**Gender**					
	Female	11.9	68.1	20.0	
	Men	16.8	66.7	16.5	
**Race**					
	White	11.1	70.9	17.9	***
	African Americans	27.8	45.1	27.1	
	Other race	25.2	47.0	27.8	
**Age groups**					
	22-39	16.4	57.6	26.0	**
	40-49	12.2	55.9	31.9	
	50-64	12.1	65.9	22.0	
	65,+	14.2	76.2	9.6	
**Enabling factors**					
**Marital status**					
	Married	11.1	74.5	14.4	**
	Not married	15.2	60.2	24.5	
**Education**					
	Less than high school	17.1	67.6	15.2	**
	High school	17.3	70.1	12.6	
	More than high school	9.6	66.3	24.1	
**Poverty Status**					
	Poor	13.7	63.7	22.6	
	Middle income	15.1	68.3	16.7	
	High income	11.2	71.0	17.8	
**Insurance**					
	Private	12.3	72.6	15.0	*
	Public	13.8	60.5	25.6	
	Uninsured	16.3	56.3	27.4	
**Need factors**					
**Perceived physical health**					
	Excellent/very good	13.2	72.6	14.2	*
	Good	11.3	72.7	16.0	
	Fair/poor	14.3	61.5	24.2	
**Perceived mental health**					
	Excellent/very good	12.2	75.6	12.1	**
	Good	11.9	70.3	17.8	
	Fair/poor	15.1	57.4	27.5	
**Anxiety**					
	Anxiety	7.4	54.3	38.2	***
	No anxiety	15.0	72.3	12.8	
**Pain**					
	Pain	11.7	68.3	19.9	
	No pain at all	20.7	62.0	17.4	
**COPD**					
	COPD	9.4	67.4	23.2	
	No COPD	14.7	67.9	17.3	
**Diabetes**					
	Diabetes	8.7	72.4	18.8	
	No diabetes	14.4	66.3	19.3	
**Heart disease**					
	Heart	10.4	74.4	15.2	
	No heart	14.2	65.1	20.7	
**Functional disability**					
	Yes	17.3	60.5	22.2	*
	No	17.9	65.3	16.9	
**Personal health practice factors**					
**BMI**					
	Under-normal weight	15.6	74.3	10.1	*
	Overweight	15.3	63.5	21.2	
	Obese	10.7	66.8	22.5	
**Smoking**					
	Current smoker	11.7	60.3	27.9	*
	Other	13.2	69.5	17.2	
**Exercise**					
	3 times per week	14.7	66.4	18.9	
	No exercise	11.9	68.7	19.4	
**External healthcare environment**					
**Metro**					
	Metro	14.4	66.9	18.7	
	Rural	7.3	71.4	21.2	

Note: Based on 647 adults, aged 21 years older with self-reported depression and Osteoarthritis who were alive during the calendar year (2008 and 2010). Asterisks represent significant group differences by depression treatment categories based on chi-square tests.

*** p < .001; ** .001 ≤ p < .01; * .01 ≤ p < .05.

Subgroup differences by type of treatment were also observed. For example, a significantly lower proportion of elderly over the age of 65 years received combination therapy (9.6%) compared to the younger age group 22–39 years (26.0%). However, a significantly higher proportion of elderly reported using antidepressants for depression treatment (76.2%) compared to the younger adults in the age group 22–39 years (57.6%). Similarly, a higher proportion of individuals with fair/poor perceived mental health (27.5%) received combination therapy compared to adults with excellent/very good perceived mental health (12.1%). Compared to those with fair/poor perceived mental health (57.4%) a higher proportion of adults with excellent/very good perceived mental health (75.6%) reported using antidepressants only.

AORs and 95% CIs from multinomial logistic regression on depression treatment among individuals with OA are summarized in Table 2. Among individuals with OA, African Americans were significantly less likely to use antidepressants as compared to whites (AOR=0.33, 95% CI=0.21, 0.51); they were also less likely to use combination therapy (AOR = 0.39, 95% CI=0.23, 0.65). Individuals with anxiety were more likely to use antidepressants only and combination therapy. Individuals with anxiety were almost 3 times (AOR = 3.52; 95% CI = 2.40, 5.15) as likely as those without anxiety to report using combination therapy; they were more likely to use antidepressants only (AOR = 1.53, 95% CI = 1.06, 2.22) compared to those without anxiety.

**Table 2:** Adjusted odds ratios and 95% confidence intervals from multinomial logistic regression on depression treatment among individuals with depression and osteoarthritis: medical expenditures panel survey, 2008 and 2010

		**Antidepressants only**			**Antidepressants & psychotherapy**		
		**AOR**	**95% CI**	**Sig**	**AOR**	**95% CI**	**Sig**
**Predisposing factors**							
**Gender**							
	Male						
	Female	1.67	[1.28,2.18]	***	1.42	[0.96,2.09]	
**Race**							
	White						
	African Americans	0.33	[0.21,0.51]	***	0.39	[0.23,0.65]	***
	Other race	0.39	[0.26,0.58]	***	0.60	[0.36,0.98]	*
**Age**							
	22-39						
	40-49	1.60	[1.09,2.34]	*	1.27	[0.82,1.98]	
	50-64	2.24	[1.46,3.42]	***	1.43	[0.90,2.26]	
	65,+	2.19	[1.40,3.43]	***	0.53	[0.28,0.98]	*
**Enabling factors**							
**Marital status**							
	Married						
	Not married	0.71	[0.54,0.95]	*	1.01	[0.68,1.49]	
**Education**							
	High school						
	Less than high school	0.68	[0.45,1.04]		0.39	[0.24,0.62]	***
	More than high school	0.89	[0.64,1.22]		0.55	[0.36,0.84]	**
**Poverty status**							
	High income						
	Poor	0.86	[0.58,1.28]		0.88	[0.55,1.42]	
	Middle income	0.76	[0.53,1.10]		0.52	[0.32,0.82]	**
**Insurance**							
	Private						
	Public	0.82	[0.58,1.17]		1.57	[0.99,2.51]	
	Uninsured	0.62	[0.40,0.95]	*	0.72	[0.43,1.21]	
**Need factors**							
**Perceived mental health**							
	Excellent/very good						
	Good	0.99	[0.72,1.35]		1.53	[0.94,2.48]	
	MH Fair/poor	0.64	[0.45,0.92]	*	1.93	[1.15,3.25]	*
**Anxiety**							
	No anxiety						
	Anxiety	1.53	[1.06,2.22]	*	3.52	[2.40,5.15]	***
**Perceived physical health**							
	Excellent/very good						
	Good	0.90	[0.61,1.34]		0.80	[0.46,1.39]	
	Fair/poor	0.84	[0.54,1.31]		0.59	[0.34,1.01]	
**Pain**							
	No pain at all						
	Pain	1.17	[0.85,1.60]		0.92	[0.61,1.39]	
**COPD**							
	No COPD						
	COPD	1.07	[0.73,1.57]		1.04	[0.69,1.57]	
**Diabetes**							
	No diabetes						
	Diabetes	1.64	[1.20,2.25]	**	1.34	[0.90,2.01]	
**Heart disease**							
	No heart disease						
	Heart disease	1.49	[1.07,2.09]	*	1.32	[0.88,1.99]	
**Functional disability**							
	No						
	Yes	0.99	[0.73,1.34]		1.39	[0.94,2.05]	
**Personal health practice factors**							
**BMI**							
	Under-normal weight						
	Overweight	1.08	[0.77,1.53]		1.13	[0.71,1.78]	
	Obese	1.31	[0.92,1.87]		1.51	[1.01,2.25]	*
**Smoking status**							
	Other						
	Current smoker	1.15	[0.77,1.72]		1.21	[0.81,1.81]	
**Exercise**							
	3 times per week						
	No exercise	0.92	[0.69,1.22]		0.94	[0.65,1.36]	
**External healthcare environment**							
**Metro**							
	Metro						
	Rural	1.28	[0.86,1.89]		1.03	[0.67,1.58]	
							

Note: Based on 647 adults, aged 21 years older with self-reported depression and Osteoarthritis who were alive during the calendar year (2008 and 2010). Asterisks represent significant group differences by type of treatment compared to the reference group based on multinomial logistic regression. The reference group for the dependent variable in the multinomial logistic regression was “No Depression Treatment”.

AOR: Adjusted odds ratio; CI: Confidence Interval; Sig: significance.

***p< .001;**.001 ≤ p<.01;*.01≤ p<.05.

Elderly individuals with OA (age 65 and older) were more likely to use antidepressants only (AOR = 2.19, 95% CI = 1.40, 3.43) compared to younger adults in the age group 22–39 years. However, they were less likely to receive combination therapy (AOR = 0.53, 95% CI = 0.28, 0.98) compared to younger adults. Similarly, adults with fair or poor perceived mental health were more likely to receive combination therapy (AOR = 1.93, 95% CI = 1.15, 3.25) compared to those with excellent or very good perceived mental health. However, they were less likely to receive antidepressants only (AOR = 0.64, 95% CI = 0.45, 0.92) compared to those with excellent or very good or good perceived mental health.

Women with OA were more likely to use antidepressants only as compared to men (AOR = 1.67, 95% CI = 1.28, 2.18). Significant differences were not observed between genders for combination therapy. Adults with chronic conditions such as diabetes (AOR = 1.64, 95% CI 1.20, 2.25) and heart disease (AOR = 1.49, 95% CI = 1.07, 2.09) were more likely to receive antidepressants only compared to those without diabetes or heart disease. Again, statistically significant associations were not observed between combination therapy and type of chronic conditions. Adults with obesity were more likely to receive combination therapy (AOR = 1.51, 95% CI = 1.01, 2.25) compared to those with underweight/normal BMI category. However, no significant differences were observed for antidepressants use only between BMI categories.

## Discussion

The current study set out to examine patterns of depression treatment among individuals with OA and depression using a nationally representative data on non-institutionalized civilian US population. In the study sample of adults with OA, 13.0% did not receive any treatment for depression and 87% reported antidepressant use (either alone or as combination therapy). The rate of depression treatment found in this study was higher than that reported elsewhere. For example, a study that examined the patterns of depression treatment trends from 1998 through 2007 among all adults reported an antidepressant use of 75.3% in 2007 [26]. However, the results cannot be directly compared due to differences in study population and time period.

Approximately, one in five individuals with OA and depression used combination therapy for depression treatment. Given the beneficial effect of combination therapy to provide relief from depressive symptoms as well as pain one would expect a higher percentage of individuals with arthritis to be treated with combination therapy. Future research needs to address the barriers to combination therapy for depression care in this population. However, the rate of psychotherapy use found in this study is consistent with published literature in which 24% individuals used psychotherapy in 2005 from office-based psychiatrists [27]. On the contrary, another study that analyzed national trends in outpatient psychotherapy found lower rates of psychotherapy use estimated to be 10.5% in 2007 [28]. Rate of psychotherapy use in the study sample may not be directly comparable as the treatment group included both antidepressants and psychotherapy.

While this study did not analyze the reasons for no treatment, it is evident from other studies that cost, side effects, severity of depression, stigma, patient-provider relationship, lack of previous family history of depression, fear of referral to the psychiatrist are barriers to depression treatment [29,30]. It is also plausible individuals with OA may rely on alternative and complementary therapies such as yoga that have been found to be effective in treating depression [31] and therefore, this study may have over-estimated rate of non-treatment.

Although evidence supports the use of antidepressants for pain among individuals with major chronic pain conditions [24], an interesting finding in this study was the absence of a relationship between pain and antidepressants use in both bivariate and multivariate models. Absence of the relationship between pain and antidepressant use needs to be interpreted with caution because of a very small sample size of adults without pain.

The study findings with respect to racial disparities in depression treatment among individuals with OA are consistent with the existing literature. For example, African Americans were less likely to receive antidepressants and combination therapy for depression compared to whites. Existing studies have suggested that African Americans were less likely to accept antidepressants and counseling as compared to other racial/ethnic subgroups due to socio economic status, access to care and patient preferences [32-34].

This study found greater likelihood of antidepressants use and lower likelihood of psychotherapy among elderly compared to younger adults. These findings are consistent with evidence from published literature that suggests an association between increasing age and decreasing odds of receiving psychotherapy [35,36]. As mentioned in the introduction combination therapy has been found to be effective in reducing pain and improving depressive symptoms among older adults [4]. Given the beneficial effects of combination therapy for the elderly, the study findings suggest that current depression care may not be optimal for elderly with OA. While this study did not explore the reasons for lower rates of psychotherapy among elderly with OA, it can be speculated that elderly may not receive psychotherapy due to access barriers in the form of high co-payments related to psychotherapy [37] or cognitive impairment [36]. Future studies are needed to explore reasons for low uptake of combination therapy so that interventions can be tailored to promote optimal therapy for depression care among elderly with depression and OA.

In this study combination therapy for depression was more likely in individuals with anxiety compared to individuals with depression and without anxiety. It is well documented that anxiety often co-occurs with depression in adults with arthritis [1]. In this study, nearly 25% of individuals with OA and depression also reported anxiety disorders. Although treatment for comorbid depression and anxiety differ by the type of anxiety disorder, cognitive-behavioral therapy (CBT), has well-documented efficacy for both depression and anxiety disorders [38].

The statistically significant association between obesity and combination therapy is also worth noting. Obesity and depression have bi-directional relationship and they often occur together [39], however, there have been no studies on combination therapy among individuals with obesity and depression. In a pilot study it was documented that individuals who were provided CBT for depression along with evidence-based behavioral treatment for obesity achieved a clinically significant reduction in depressive symptoms in 16 weeks [40]. Therefore, it is plausible that those with obesity and depression may receive treatments that combine psychotherapy for depression to achieve better clinical outcomes.

Findings from this study need to be interpreted in the light of its strengths and limitations. Strengths include a nationally representative survey, availability of a comprehensive list of variables that may be associated with depression treatment. Furthermore, prescription drug and psychotherapy information allowed us to categorize no depression treatment as well as type of depression treatment. However, there are some limitations. All measures were self-reported and subject to recall bias. Only general psychotherapy was measured and distinctions between types of psychotherapies could not be used, which may be important in determining appropriate care. Patient preferences and use of alternative and complementary medicine for depression treatment were not included. Therefore, the study could not identify the reasons for lack of depression treatment among individuals with OA.

## Conclusions

Despite these limitations, this study added to the emerging nascent literature on depression treatment patterns among individuals with chronic physical conditions, specifically OA. It also highlighted many subgroup differences in likelihood of treatment and type of depression treatment. Although combination therapy is proven effective among individuals with OA, some subgroups such as the elderly and those with chronic illnesses (example: diabetes and heart disease) did not report receiving combination therapy. Future research needs to evaluate barriers to depression care among African Americans and challenges to combination therapy for some subgroups of adults with OA. In addition, further studies need to be conducted as to whether lack of depression treatment is associated with poor health outcomes such as functional status among those with OA.

## Abbreviations

RA: Rheumatoid arthritis; OA: Osteoarthritis; IMPACT: Improving Mood Promoting Access to Collaborative Treatment; COPD: Chronic obstructive pulmonary disease; MEPS: Medical Expenditure Panel Survey; AHRQ: Agency for Healthcare Research and Quality; ICD-9-CM: International Classification of Diseases, 9th Edition, Clinical Modification; BMI: Body mass index.

## Competing interests

The views expressed in this academic research paper are those of the authors and do not reflect the official policy or position of West Virginia University (WVU) or any other affiliated organizations.

## Authors’ contributions

1) PA reviewed the literature, facilitated data management, work on data analysis, drafted the manuscript and incorporated suggestions from co-authors in successive iterations. 2) XP advised on data analysis, interpreted findings and provided input on successive iterations of the manuscript. 3) US designed the study, supervised data analysis, interpreted findings and provided input on successive iterations of the manuscript. All authors read and approved the final version of the manuscript.

## Pre-publication history

The pre-publication history for this paper can be accessed here:

http://www.biomedcentral.com/1471-244X/13/121/prepub
